# Sensor Fusion for Power Line Sensitive Monitoring and Load State Estimation

**DOI:** 10.3390/s23167173

**Published:** 2023-08-14

**Authors:** Manuel Schimmack, Květoslav Belda, Paolo Mercorelli

**Affiliations:** 1 Institute for Production Technology and Systems, Leuphana University of Lueneburg, Universitätsallee 1, D-21335 Lueneburg, Germany; 2 The Czech Academy of Sciences, Institute of Information Theory and Automation, Department of Adaptive Systems, Pod Vodárenskou věží 4, CZ-18200 Prague, Czech Republic; belda@utia.cas.cz

**Keywords:** soft sensing, fault detection, state estimation of electrical systems, transformers

## Abstract

This paper deals with a specific approach to fault detection in transformer systems using the extended Kalman filter (EKF). Specific faults are investigated in power lines where a transformer is connected and only the primary electrical quantities, input voltage, and current are measured. Faults can occur in either the primary or secondary winding of the transformer. Two EKFs are proposed for fault detection. The first EKF estimates the voltage, current, and electrical load resistance of the secondary winding using measurements of the primary winding. The model of the transformer used is known as mutual inductance. For a short circuit in the secondary winding, the observer generates a signal indicating a fault. The second EKF is designed for harmonic detection and estimates the amplitude and frequency of the primary winding voltage. This contribution focuses on mathematical methods useful for galvanic decoupled soft sensing and fault detection. Moreover, the contribution emphasizes how EKF observers play a key role in the context of sensor fusion, which is characterized by merging multiple lines of information in an accurate conceptualization of data and their reconciliation with the measurements. Simulations demonstrate the efficiency of the fault detection using EKF observers.

## 1. Introduction

Today, there are many low-power household and medical appliances containing simple inverters or mutual inductors. There are also many devices in industry, e.g., drives, where the situation is similar but with high performance. In all of these applications, the effort is to replace conventional sensors with soft ones and to ensure fault-free operation. The following section provides an overview of the current state of possible methods and their implementation in specific fault detection applications. This contribution focuses particularly on mathematical methods useful for soft sensing and fault detection. Moreover, EKF observers play a key role in the context of sensor fusion, which is characterized by a modern sensor’s structure for merging multiple lines of information in a more accurate conceptualization of data and their reconciliation with the measurements. In this context of conceptualization and reconciliation of the measured data, sensor fusion represents one of the crucial key points in fault detection.

### 1.1. Fault Detection

Due to the need to guarantee safety conditions and continuity of service in technical systems, detecting faults is one of the most challenging and important tasks in control systems and monitoring; see [1,2]. The importance of this task is not just limited to technical systems; it is important and crucial in any field of application. In this context, a recent proposed approach in [3] investigates a general method that uses only system input/output data collected via sensor networks. It proposes a new method to analyze the residual signals, which are combined with the Hellinger distance to improve the performance of the method. The method proposed in [3] takes into particular consideration the presence of noise. In fact, in the presence of strong noise, detecting faults becomes a difficult task. In the context of safety, the importance of the fault detection is clearly stated by the presence of norms. Methods of applying, designing, deploying, and maintaining safety-related systems are described within the international standards IEC 61508 [4] (Functional Safety of Electrical/Electronic/Programmable Electronic Safety-related Systems). Thanks to these standards, a large number of compliant products and processes have been created, such as automotive safety components, medical devices, sensors, actuators, diving equipment, and process controllers. In particular, IEC 61508 defines through ISO 26262 [5] a series of standards that are dedicated to automotive components and are used in a more general context of functional safety to suppress and eliminate undue risks caused by improper behavior of electrical/electronic systems. Recent publications include [6], in which redundant virtual sensors were applied to guarantee continuity in service. In [7], the dual concept of virtual sensors using EKFs was used to maintain continuity of service in DC/DC systems. This recent literature indicates the importance of this topic and the need to explore new solutions for evolving products and systems. Fault detection is a fundamental procedure for obtaining fault-tolerant systems. Such systems continue to function correctly even if a certain number of errors occur during their execution. The development and verification of fault-tolerant systems requires scientific methodology that includes modeling methods, design patterns for fault-tolerance methods, and ready-made algorithms. In design, it is important to recognize faults and evaluate whether they are dangerous. Different measures can be taken into account to assess their nature. Faulty components can be replaced with ready-made new components in standby mode, or the system can be shut down in an irreparable state.

As stated in the seminal work [8], many contributions have been proposed for state observation and monitoring. They were collected in [9]. The Kalman filter (KF) can be used as an asymptotic observer [10], proven by the direct Lyapunov method to estimate deflections. It can be used in fault detection to monitor states, components, and working points of machines and complex plants and can serve as an auxiliary observer of a supervisory system with higher algorithms and logic elements. In the last few years, many different contributions have appeared in all technical fields. The extended Kalman filter (EKF) was proposed for the parameter estimation of induction motors and for the speed and position estimation of brush-less DC motors [11].

Fault detection is also a challenge in terms of fault isolation. In this context, observer-based techniques have been applied in many technical fields, such as sensor fault detection for induction motors [12], aircraft engine fault diagnostic systems [13], and air conditioning [14], based on a combination of ARX structure and EKF. To achieve functional safety in terms of a fault-tolerant system, the term “hardware redundancy” often means redundant sensors and/or redundant actuators connected in standby to the system. This method is very expensive. In general, in fault detection, observers as virtual sensors and virtual actuators are used to detect faults and errors and to replace the faulting component, if possible, using a “virtual one”. Thus, observers as virtual sensors can be successfully used for monitoring and support of fault detection structures without using direct sensors as fault detectors.

The determination of inrush and internal fault currents in transformers is an important feature of the transformer protection scheme proposed in [15,16,17]. In particular, in [15], a classification, in the sense of a discrimination, of internal fault currents and the phenomenon of inrush currents in a transformer is realized by using an EKF algorithm based on the monitoring of current and resistance variation on the primary winding. In [16], a fault diagnosis in a permanent magnet synchronous generator (PMSG) is proposed and, when the fault appears, an EKF and unscented Kalman filter (UKF) are used to detect the percentage and the place of the fault. In [17], high-order compensation topology integration for high-tolerant wireless power transfer is proposed, in which description and comparison of highly flexible compensation topologies, including integration methods and relative control strategies for high misalignment-tolerant wireless power transformer systems, are proposed. All of these contributions indicate the importance of the topic as well as the variety of the possible problems that can occur and their possible solutions. A current sensor fault-tolerant control strategy based on criteria markers is presented for permanent magnet synchronous motor drive systems in [18,19]. EKF in a learning-based approach to fault detection and classification in three-phase power transformers is presented in [20].

Fault detection and diagnosis (FDD) is another challenge in power system protection. Its comprehensive review and classification for the last three decades is provided in [21]. FDD for a robust design is shown in [22], where the combination of different techniques, including state estimation, statistical, spectral analysis, model- and signal-based approaches, and deep learning, is discussed. A review of the methods of fault detection, classification, and location for transmission lines and distribution systems is provided in [23], where the time interval for fault detection is part of the discussion. Reactions, fault detection, challenges, and future prospects of power transformer insulation systems are discussed in [24].

Identification of short circuits in low-voltage networks using a tolerant locus curve criterion is presented in [25]. The criterion is independent of the power factor and initial current. It indicates the short circuit if its values are outside of the tolerance locus curve. The design and evaluation of a hybrid system for fault detection and prediction in electrical transformers is shown in [26]. Diagnosis of interturn faults of voltage transformers using excitation current and phase differences is discussed in [27].

In this paper, we focus on fault detection using an EKF structure for sensor fusion based on original principles, as summarized in Section 1.2 on our paper’s contributions.

### 1.2. Contribution

This paper proposes fault detection using extended Kalman filters for sensor fusion. Contributions for the purposes of fault detection are summarized as follows:

- The original principle of a specifically adapted EKF as observer that estimates the fault condition of the power line of electrical health device management (fluctuation of the mains voltage of the power line in Europe is in the tolerance of 230±23V at a mains frequency of 50±0.2Hz; see DIN IEC 60038 [28].
- The original principle of another EKF for a state estimation of the secondary galvanic decoupled side of a two-winding transformer and the electrical load resistance $R_{L}$.

The paper is organized as follows. The problem of fault detection is formulated in Section 2. Section 3 deals with two specific EKFs for a mutual inductor with load and for power line conditions. An experimental setup for demonstration of the proposed approach is described in Section 4. Finally, Section 5 shows and discusses the obtained results. The paper also contains Appendix A, in which there is a printout of the EKF procedures.

## 2. Problem Formulation

The fault detection problem is to create a detectable signal by which the fault can be detected. As a target application, we investigate a specific two-winding transformer (mutual inductor) connected to one phase of the power line and the detection of specific faults, or electrical limit states, immediately upon occurrence of the fault. The model of the mutual inductor is described in Section 2.1. The complete transformer system including fault detector is described in Section 2.2. The detector based on the software principle using EKFs is described in Section 3.

### 2.1. Mutual Inductor and Transformer

The mutual inductance model is an alternative approach to describe the physical properties of a transformer, such as its magnetic permeability or dimensions. This method is not very popular in the power systems community but has gained consensus in systems theory. In it, and particularly in connection with the adaptive estimation of parameters that characterize mutual inductance, such as the coupling coefficient *K*, the mutual inductance approach is preferred for its flexibility and straightforward structure.

Although the mutual inductance approach does not directly emphasize the physical aspects of the transformer, it guarantees an efficient description of the related physical phenomenon as follows. An important parameter to be estimated is the coupling coefficient $K=\frac{\phi_{m}}{\phi_{m}+\phi_{1}}$, in which $\phi_{m}$ represents the common flux (core flux) and $\phi_{1}$ the primary stray flux, which will be considered variable over time *t*.

Recent publications, such as [29], look at the optimal impedance on the secondary side of the “transformer” as well as improving transfer efficiency. It is necessary to know the coupling coefficient. A coupling coefficient observer method based on KF is proposed to adapt the optimal impedance controller on the secondary side. The mutual inductance approach is usually preferred in the asynchronous motor model using two combined EKFs for state and parameter estimation of the induced motor.

**Figure 1:**
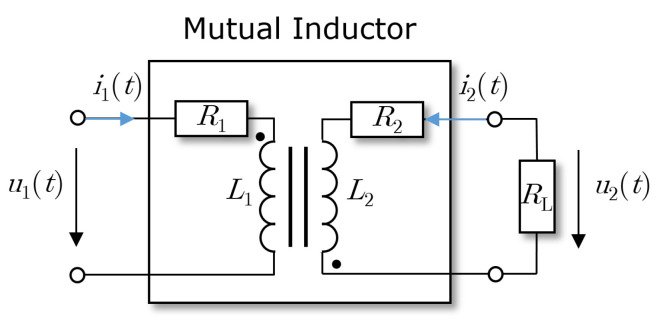
Scheme of mutual inductance with electrical load resistance $R_{L}\left(t\right)$.

A two-winding transformer as the mutual inductor and its equivalent components, with the electrical series resistance $R_{1}$ and the inductance $L_{1}$ of the primary winding, are shown in Figure 1. The secondary winding is represented by the electrical resistance $R_{2}$ and the inductance $L_{2}$ together with the electrical load resistor $R_{L}\left(t\right)$.

The voltage distribution of the primary winding of the mutual inductance is as:

(1) $$u_{1}\left(t\right)=R_{1}\;i_{1}\left(t\right)+L_{1}\frac{d\;i_{1}\left(t\right)}{d\;t}+M\;\frac{d\;i_{2}\left(t\right)}{d\;t},$$

where $i_{1}$(*t*) and $i_{2}$(*t*) are the currents across the primary and secondary winding, respectively. The dynamics of the voltage distribution across the secondary winding are as follows:

(2) $$u_{2}\left(t\right)=L_{2}\frac{d\;i_{2}\left(t\right)}{d\;t}+M\;\frac{d\;i_{1}\left(t\right)}{d\;t}+R_{2}\;i_{2}\left(t\right),$$

and for mutual inductance, *M* is defined as follows:

(3) $$M=K\;\sqrt{L_{1}\;L_{2}},\;\;M^{2}=K^{2}\;L_{1}\;L_{2},$$

where *K* is the inductive coupling coefficient. Thus, the dynamics of voltage distribution are described by Equations (1)–(3). Then, the current $i_{1}$(*t*) of the primary winding is:

(4) $$\frac{d\;i_{1}\left(t\right)}{d\;t}=\frac{1}{L_{1}\left(1-K^{2}\right)}\left(u_{1}\left(t\right)-R_{1}\;i_{1}\left(t\right)+\frac{M}{L_{2}}\;R_{L}\;i_{2}\left(t\right)+\frac{MR_{2}}{L_{2}}\;i_{2}\left(t\right)\right),$$

and the current $i_{2}\left(t\right)$ for the secondary winding is:

(5) $$\frac{d\;i_{2}\left(t\right)}{d\;t}=\frac{1}{L_{2}\left(1-K^{2}\right)}\left(-R_{L}\;i_{2}\left(t\right)-R_{2}\;i_{2}\left(t\right)-\frac{M}{L_{1}}\;u_{1}\left(t\right)-\frac{M\;R_{1}}{L_{1}}\;i_{1}\left(t\right)\right).$$

Time derivatives for the proposed inductive coupling coefficient and for the electrical load resistance of the secondary side are assumed to be:

(6) $$\begin{array}{cc} \frac{d\;K}{d\;t} & =0,\;\;\;\;\;\;\;\frac{d\;R_{L}}{d\;t}=0. \end{array}$$

The model of the two-winding transformer in Equations (3)–(6) uses voltage distribution from Equations (1)–(3) mentioned above. The model represents the system of differential equations in normal form, i.e., the first time, derivative terms are located on one side and other algebraic terms on the opposite side. This form is suitable for matrix notation and state-space formulations. This feature will be individually used to our advantage in subsequent sections on EKF methods.

### 2.2. Transformer System

**Figure 2:**
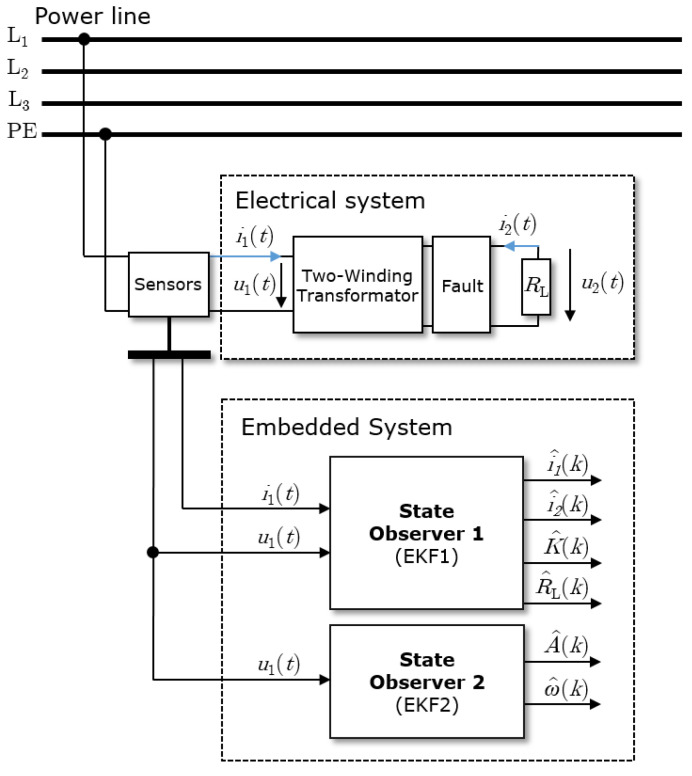
System with the observers and the measured input voltage $u_{1}$(*t*) and current $i_{1}$(*t*) together with the electrical system (mutual inductor with electrical load resistance $R_{L}$).

A structural scheme of the considered electrical system and the proposed observer strategy is depicted in Figure 2. The measured inputs are voltage $u_{1}$(*t*) and current $i_{1}$(*t*) of the power line. Two EKFs are designed and implemented to reduce the calculation load through the reduction of the dimensions of the algorithm matrices. The aim is to estimate the state of the mutual inductance together with the load resistance $R_{L}$ and to detect harmonics or faults in the power line. The proposed structure has some similarities to the adaptive KF in [1], where it is used for the approximation of the derivative.

## 3. EKF Methods

EKF methods are usually applied to state-space estimation of a class of nonlinear systems. A structural limit of this approach is that EKF does not guarantee either the global convergence or the optimality of the estimation. Here, they are applied to two specific subsystems to estimate values of unmeasured electric quantities for both primary and secondary winding.

### 3.1. State Observer for the Mutual Inductance and Load–EKF1

The EKF1 is utilized as an observer to estimated the state of the mutual inductor and the electrical load resistance $R_{L}\left(t\right)$. It is necessary for the EKF1 to express Equations (4) and (5) in a state-space representation with the measurement matrix:

(7) $$\begin{array}{cc} x(t) & ={\left[\begin{array}{cccc} i_{1}\left(t\right) & i_{2}\left(t\right) & K(t) & R_{L}\left(t\right) \end{array}\right]}^{T}\;\;,\;\;\;\;\dot{x}\left(t\right)=f\left(x\left(t\right),\;u_{1}\left(t\right)\right), \end{array}$$

(8) $$\begin{array}{cc} f(x(t),u_{1}\left(t\right)) & =\left[\;\;\;\begin{array}{c} \frac{1^{{}^{{}^{{}^{{}^{}}}}}}{L_{1}\;\left(1-K{\left(t\right)}^{2^{{}^{}}}\right)}\;\left(u_{1}\left(t\right)-R_{1}\;i_{1}\left(t\right)+\frac{M_{{}_{}}}{L_{2}^{{}^{}}}-R_{L}\left(t\right)\;i_{2}\left(t\right)+\frac{M\;R_{2}}{L_{2}^{{}^{}}}\;i_{2}\left(t\right)\right)\\ \frac{1}{L_{2}\;\left(1-K{\left(t\right)}^{2^{{}^{}}}\right)}\;\left(\;-R_{L}\left(t\right)\;i_{2}\left(t\right)-R_{2}\;i_{2}\left(t\right)-\frac{M_{{}_{}}}{L_{1}^{{}^{}}}u_{1}\left(t\right)-\frac{M\;R_{1}^{{}^{{}^{}}}}{L_{1}^{{}^{}}}\;i_{1}\left(t\right)\right)\\ K(t)\\ \;R_{L}{\left(t\right)}^{{}^{{}^{}}} \end{array}\;\;\;\right]. \end{array}$$

Using the Euler discretization with the sampling time $T_{s}$ and the discrete model of the dynamics of the electrical current, the coupling and the voltage across the secondary winding can be expressed by the following discrete time equations:

(9) $$\begin{array}{cc} i_{1,\;k} & =i_{1,\;k-1}+\frac{T_{s}}{L_{1}\left(1-K^{2}\right)}\left(u_{1,\;k-1}-R_{1}\;i_{1,\;k-1}+\frac{M}{L_{2}}-R_{L}\;i_{2,\;k-1}+\frac{M\;R_{2}}{L_{2}}\;i_{2,\;k-1}\right) \end{array}$$

(10) $$\begin{array}{cc} i_{2,\;k} & =i_{2,\;k-1}+\frac{T_{s}}{L_{2}\left(1-K^{2}\right)}\left(-R_{L}\;i_{2,\;k-1}-R_{2}\;i_{2,\;k-1}-\frac{M}{L_{1}}u_{1,\;k-1}-\frac{MR_{1}}{L_{1}}\;i_{2,\;k-1}\right) \end{array}$$

where (9) and (10) can be given in a state-space form inside the Kalman filter algorithm as:

(11) $${\hat{x}}_{k-1}={\left[\;i_{1,\;k-1}\;\;i_{2,\;k-1}\;\;K_{k-1}\;\;R_{L,\;k-1}\;\right]}^{T},\;\;\;{\hat{x}}_{k}^{-}=f\left({\hat{x}}_{k-1},\;u_{1,\;k-1}\right).$$

This deterministic dynamic model is expressed by the following matrix notation with one time-step subscript ${\;}_{\;k\;-\;1}$, which belongs to all the time-varying parameters of the matrix:

(12) $$\;\;\;\;\;\underset{{\left[f_{1},\;f_{2},\;f_{3},\;f_{4}\right]}^{T}}{\underset{︸}{f({\hat{x}}_{k-1},\;u_{1,\;k-1})}}={\left[\;\;\begin{array}{c} i_{1}+\frac{T_{s}^{{}^{{}^{{}^{{}^{}}}}}}{L_{1}\left(1-K^{2^{{}^{}}}\right)}\;\left(u_{1}-R_{1}\;i_{1}+\frac{K\sqrt{L_{1}L_{2}}}{L_{2}^{{}^{}}}\;R_{L}\;i_{2}+\frac{K\sqrt{L_{1}L_{2}}}{L_{2}^{{}^{}}}\;R_{2}\;i_{2}\right)\\ i_{2}+\frac{T_{s}}{L_{2}\;\left(1-K^{2^{{}^{}}}\right)}\;\left(\;-R_{L}\;i_{2}-R_{2}\;i_{2}-\frac{K\sqrt{L_{1}L_{2}}}{L_{1}^{{}^{}}}\;u_{1}-\frac{K\sqrt{L_{1}L_{2}}}{L_{1}^{{}^{}}}\;R_{1}\;i_{1}\right)\\ K\\ R_{L}^{{}^{{}^{{}^{}}}} \end{array}\;\;\right]}_{{}^{{}^{\;\;k-1}}}\;\;\;\;\;\;\;\;\;\;.\;\;\;\;\;\;\;\;\;\;$$

The discrete Jacobian matrix is defined, using the similar subscript ${\;}_{\;k\;-\;1}$, as follows:

(13) $$\begin{array}{cc} \;\;\;\;J_{k}\; & =\;{\left[\begin{array}{cccc} \frac{\partial \;f_{1}}{\partial \;i_{1}} & \frac{\partial \;f_{1}}{\partial \;i_{2}}\; & \frac{\partial \;f_{1}}{\partial K_{}} & \frac{\partial \;f_{1}}{\partial R_{L}}\\ \frac{\partial \;f_{2}}{\partial \;i_{1}} & \frac{\partial \;f_{2}^{{}^{{}^{{}^{}}}}}{\partial \;i_{2}}\; & \frac{\partial \;f_{2}^{{}^{{}^{{}^{}}}}}{\partial K_{}} & \frac{\partial \;f_{2}^{{}^{{}^{{}^{}}}}}{\partial R_{L}}\\ \frac{\partial \;f_{3}^{{}^{{}^{{}^{}}}}}{\partial \;i_{1}} & \frac{\partial \;f_{3}^{{}^{{}^{{}^{}}}}}{\partial \;i_{2}}\; & \frac{\partial \;f_{3}^{{}^{{}^{{}^{}}}}}{\partial K_{}} & \frac{\partial \;f_{3}^{{}^{{}^{{}^{}}}}}{\partial R_{L}}\\ \frac{\partial \;f_{4}^{{}^{{}^{{}^{}}}}}{\partial \;i_{1}} & \frac{\partial \;f_{4}^{{}^{{}^{{}^{}}}}}{\partial \;i_{2}}\; & \frac{\partial \;f_{4}^{{}^{{}^{{}^{}}}}}{\partial K_{}} & \frac{\partial \;f_{4}^{{}^{{}^{{}^{}}}}}{\partial R_{L}} \end{array}\right]}_{{}_{{}_{{}_{{}_{{}_{{}_{}}}}}}^{{}^{\;k-1}}}\\ \;\;\;\; & =\;{\left[\;\;\;\;\begin{array}{cccc} 1\;-\;\frac{R_{1}\;T_{s_{{}_{}}}}{L_{1}\;{\left(1-K^{2}\right)}^{{}^{{}^{}}}}\;\;\; & \;\;\;\frac{K\;T_{s}\;{\left(R_{L}\;+R_{2}\right)}_{{}_{{}_{}}}}{{\sqrt{L_{1}L_{2}}}^{{}^{}}\;{\left(1-K^{2}\right)}_{{}_{{}_{{}_{{}_{{}_{\;}}}}}}}\;\;\; & \;\;\;{\frac{\left(1-3K-K^{2}\right)T_{s}\left(R_{L}\;+R_{2}\right)\;i_{2}}{{\sqrt{L_{1}L_{2}}}^{{}^{}}{\left(1-K^{2}\right)}^{2}}}_{{}_{{}_{{}_{{}_{{}_{\;}}}}}}\;\;\; & \;\;\;\;\;\frac{K\;T_{s}\;i_{2_{}}}{\sqrt{L_{1}L_{2}}\;{\left(1-K^{2^{{}^{}}}\right)}_{{}_{{}_{{}_{{}_{{}_{\;}}}}}}}\\ \frac{-K\;Ts\;R_{1}}{\sqrt{L_{1}L_{2}}\;\left(1-K^{2}\right)}\;\;\; & \;\;\;1\;-\;\frac{T_{s}\;\left(R_{L}+R_{2}\right)}{L_{2}\;{\left(1-K^{2}\right)}^{{}^{{}^{}}}}\;\;\; & \;\;\frac{\left(-1+3K+K^{2}\right)T_{s}\left(u_{1}\;+\;R_{1}\;i_{1}\right)}{\sqrt{L_{1}L_{2}}{\left(1-K^{2}\right)}^{2^{}}}\;\;\;\; & \;\;\;\;\frac{-T_{s}\;i_{2_{}}}{L_{2}\;{\left(1-K^{2}\right)}^{{}^{{}^{}}}}\\ 0_{{}_{{}_{\;}}}^{{}^{{}^{{}^{{}^{\;}}}}} & 0 & 1 & 0\\ 0_{{}_{{}_{\;}}}^{{}^{{}^{{}^{{}^{\;}}}}} & 0 & 0 & 1 \end{array}\;\;\;\;\right]}_{{}^{{}^{\;k-1}}}\;\;\;\;\;\;\;\;\;.\;\;\; \end{array}$$

It follows for the a priori covariance estimate that:

(14) $$P_{k}^{-}=J_{k}P_{k-1}^{+}J_{k}^{T}+Q_{1}.$$

During the initial time step, $P_{k-1}^{+}=P_{0}^{+}$ is a predetermined initial value. From the second time step, $P_{k-1}^{+}$ is the previously derived a posteriori error estimate covariance. The user defines matrix $Q_{1}$, which serves as a “possible” representation of the uncertainty inherent in the model due to associated noise processes. As only a single state is measured, the denominator in Equation (15) is scalar. This leads to the use of an unconventional yet succinct notation for the Kalman gain as follows:

(15) $$k_{k}=\frac{P_{k}^{-}H_{1}}{{H_{1}}^{T}P_{k}^{-}H_{1}+r_{1}}\;.$$

The vector $H_{1}={\left[1\;0\;0\;0\right]}^{T}$ specifies which state represents the measurement innovation. In the given case, it is the electrical current $i_{1}\left(t\right)$, and the measurement uncertainty is quantified by the scalar $r_{1}$. In the update step, the a posteriori estimation of the states and the covariance of the estimation error follow from:

(16) $${\hat{x}}_{k}^{+}={\hat{x}}_{k}^{-}+k_{k}\left(i_{1}-{H_{1}}^{T}{\hat{x}}_{k}^{-}\right)$$

(17) $$P_{k}^{+}=\left(I_{4\times 4}-k_{k}{H_{1}}^{T}\right)P_{k}^{-}.$$

**Remark** **1.** *The precision of the estimation depends on the system model accuracy in the observer* EKF1* and on the precision of the output sensor. The observer can be tuned by using the covariance matrix $Q_{1}$, which states the inaccuracy of the model, and by the variance matrix $r_{1}$, which represents the variance of the output sensor; see [30,31].*

### 3.2. State Observer for the Power Line Condition– EKF2

The state observer EKF2 is utilized for harmonic detection. It is predetermined to estimate the electrical voltage amplitude A(t) and frequency ω(t) of the primary winding of the power line. For the estimation, a harmonic model is suitable as follows:

(18) $$h_{2}\left(t\right)=u_{\mathrm{EKF}2}\left(t\right)=u_{1}\left(t\right)=A\left(t\right)\;\sin\left(\omega (t)\;t\right).$$

This model includes the corresponding minimal number of physical quantities of voltage measurements $u_{1}\left(t\right)$ and considers the useful relation ϕ(t)=ω(t)t.

**Remark** **2.** *The presence of the product between A(t) and sin(ω(t)t) in Equation (18) states a non-injective function that generates a non-observable structure. In fact, it is possible to describe the measured output $u_{1}\left(t\right)$ through normally distributed physical quantities A(t) and ω(t) with means that correspond to four combinations of their positive and negative magnitudes. In order to apply this approach, it is necessary to consider positive ω(t) and positive amplitude A(t). These assumptions reflect the physical structure of the considered phenomenon.*

If frequency of the harmonic sinusoidal is constant, then $\dot{\phi }\left(t\right)=\omega$. It follows that $\dot{A}\left(t\right)=0$ if A(t) is constant. Let a model of the real world in state-space form be written as follows:

(19) $$\begin{array}{c} \left[\begin{array}{c} \dot{\phi }\left(t\right)\\ \dot{\omega }\left(t\right)\\ \dot{A}\left(t\right) \end{array}\right]=\underset{A_{F}}{\underset{︸}{\;\left[\begin{array}{ccc} 0 & 1 & 0\\ 0 & 0 & 0\\ 0 & 0 & 0 \end{array}\right]\;}}\;\left[\begin{array}{c} \phi (t)\\ \omega (t)\\ A(t) \end{array}\right]+\left[\begin{array}{c} 0\\ u_{s1}\left(t\right)\\ u_{s2}\left(t\right) \end{array}\right], \end{array}$$

where $u_{s1}\left(t\right),u_{s2}\left(t\right)$ are white noise variables and $A_{F}$ is a matrix of the system dynamics. The signals are under continuous disturbance, modeled by process noise matrix $Q_{2}$ as:

(20) $$\begin{array}{c} Q_{2}=\left[\begin{array}{ccc} 0 & 0 & 0\\ 0 & \Phi_{s1} & 0\\ 0 & 0 & \Phi_{s2} \end{array}\right], \end{array}$$

in particular, with the diagonal elements $\Phi_{s1}$ and $\Phi_{s2}$ indicating the corresponding variances. Now, the system can be discretized using the forward Euler method as follows:

(21) $$\begin{array}{cc} \dot{\phi }\left(t\right) & =\omega \left(t\right)\Rightarrow \phi_{\;k}-\phi_{\;k-1}=T_{s}\;\omega_{\;k-1}, \end{array}$$

and then Equation (19) in its discrete form is:

(22) $$\begin{array}{cc} \left[\begin{array}{c} \phi_{\;k}\\ \omega_{\;k}\\ A_{\;k} \end{array}\right] & =\left[\begin{array}{ccc} 1 & T_{s} & 0\\ 0 & 1 & 0\\ 0 & 0 & 1 \end{array}\right]\left[\begin{array}{c} \phi_{\;k-1}\\ \omega_{\;k-1}\\ A_{\;k-1} \end{array}\right]+\left[\;\begin{array}{c} 0\\ u_{s1,\;k-1}\\ u_{s2,\;k-1} \end{array}\;\right], \end{array}$$

where the Jacobian matrix $H_{2}$ of the output in (18) is as follows:

(23) $$\begin{array}{cc} \frac{du_{1,\;k}}{d\phi_{\;k}} & =A_{\;k}\;\cos\left(\phi_{\;k}\right),\;\frac{du_{1,\;k}}{d\omega_{\;k}}=0,\;\frac{du_{1,\;k}}{dA_{\;k}}=\sin\left(\phi_{\;k}\right), \end{array}$$

(24) $$\begin{array}{cc} H_{2} & =\left[\begin{array}{ccc} A_{\;k}\;\cos\left(\phi_{\;k}\right) & 0 & \sin(\phi_{\;k}) \end{array}\right]. \end{array}$$

Thus, the estimation of the amplitude A(t) and phase ϕ(t) of voltage $u_{1}\left(t\right)$ can be provided.

**Remark** **3.** *The presence of the even function $\cos(\phi_{\;k})$ in the Jacobian in Equation (24) generates a Jacobian matrix, which states a non-injective transformation and which reflects again the unobservability aspect mentioned in Remark 2. As already explained in Remark 2, in order to apply this approach, it is necessary to consider only positive values of amplitude and positive value phase.*

### 3.3. Observability Analysis

To investigate observability, a test related to “local weak observability” using Lie derivative structures is presented in the literature. The concept is strictly connected to the concept of the distinguishability in finite time [32]. For more details on the local weak observability test for the EKFs, see the Appendix with the created MATLAB R2021a^®^ code. A nonlinear system,

$$\begin{array}{c} \;\;\;\;\;\;\left\{\;\begin{array}{c} \dot{x}\left(t\right)=f\left(x(t),u_{1}\left(t\right)\right)\\ y(t)=h(x(t)) \end{array},\right. \end{array}$$

is observable if the following expression holds:

(25) $$\begin{array}{cc} \mathrm{rank}(O(x(t)))=\; & \mathrm{rank}\;\left[\begin{array}{c} O_{0}\left(x(t)\right)\;\;\;O_{1}\left(x(t)\right)\;\;\;O_{2}\left(x(t)\right)\;\;\;\cdots \;\;\;\;\;O_{n-1}\left(x(t)\right) \end{array}\right]=n,\;\; \end{array}$$

where O(x(t))

(26) $$\begin{array}{cc} \; & =\;\left[\begin{array}{c} \;\;\;{\left(\frac{\partial }{\partial x_{{}_{{}_{{}_{}}}}}\;h\left(x\right)\right)}^{T}\;\;\left(\frac{\partial }{\partial x_{{}_{{}_{{}_{}}}}}\;O_{0}\left(x\right)\;f{\left(x,\;u_{1}\right)}^{T}\right)\;\;{\left(\frac{\partial }{\partial x_{{}_{{}_{{}_{}}}}}\;O_{1}\left(x\right)\;f\left(x,\;u_{1}\right)\right)}^{T}\cdots {\left(\frac{\partial }{\partial x_{{}_{{}_{{}_{}}}}}\;O_{n-1}\left(x\right)\;f\left(x,\;u_{1}\right)\right)}^{T} \end{array}\;\;\;\right]\\ \; & =\;\left[\begin{array}{c} \;\;\;{\left(\frac{\partial }{\partial x_{{}_{{}_{{}_{}}}}}h\left(x\right)\right)}^{T}{\left(\frac{\partial }{\partial x_{{}_{{}_{{}_{}}}}}L_{f}^{}\;h\left(x\right)\right)}^{T}{\left(\frac{\partial }{\partial x_{{}_{{}_{{}_{}}}}}L_{f}^{2}\;h\left(x\right)\right)}^{T}\cdots {\left(\frac{\partial }{\partial x_{{}_{{}_{{}_{}}}}}L_{f}^{n-1}\;h\left(x\right)\right)}^{T}\;\;\; \end{array}\right]\;\;\;, \end{array}$$

with $L_{f}^{0}\;h\left(x\right)$ standing for the scalar function h(x), $\frac{\partial }{\partial x}\;\left(L_{f}^{0}\;h\left(x\right)\right)=\frac{\partial }{\partial x}\;h\left(x\right)$ for the Jacobian of h(x), and $L_{f}\;h\left(x\right)=L_{f}^{0}h\left(x\right)\cdot f\left(x,\;u_{1}\right)=\frac{\partial }{\partial x_{{}_{{}_{}}}}\;\left(h\left(x\right)\right)\cdot f\left(x,\;u_{1}\right)$ for a scalar function given by the Jacobian of h(x) scalar, multiplied by the field $f(x,\;u_{1})$. In general, it can be written as follows:

(27) $$L_{f}^{m}\;h\left(x\right)=L_{f}^{m-1}\;h\left(x\right)\cdot f\left(x,\;u_{1}\right),\;\;\;\forall \;m\in N;$$

note that $L_{f}^{m}\;h\left(x\right)=L_{f}^{m-1}\;h\left(x\right)$ is a scalar function and, according to [33], it can be given as:

(28) $$L_{f}\;h\left(x\right)=\sum_{i=1}^{n}\;\frac{\partial \;h(x)}{\partial x_{i}}\;f\left(x,\;u_{1}\right),$$

where index *i* represents the components of the field, which are equal to the number of states.

The first idea is to measure two state variables, for instance, the currents $i_{1}\left(t\right)$ and $i_{2}\left(t\right)$. In this case, the system results in being observable. In fact, it is possible to see that it is easy to find a nonzero determinant among 70 determinants, which are obtained for the observability test in accordance with the binomial expression:

(29) Nd=pn=p!n!(p−n)!,

where $N_{d}$ represents a number of possible determinants to be checked, *n* is the number of columns, and *p* is the number of rows of the local weak observability matrix.

In the presented application, it is assumed that current $i_{2}\left(t\right)$ is not available to be measured. In this case, as explained in Section 3, the measured state is current $i_{1}\left(t\right)$; see Figure 2 for a graphical visualization of the state observer. The test stated by Equation (26) is not satisfied; see Appendix for details. Nevertheless, Equation (26) represents a sufficient condition and, in this sense, no conclusion about the local weak observability of the system can be drawn.

Simulation results obtained with the selected tuning elements of the EKF1 show that the local weak observability of the considered phenomenon, which is taken into consideration, is guaranteed. More in depth, in Equation (12), if just current $i_{1}\left(t\right)$ is measured, then n=4, which is the number of the states or number of columns, including the extended states, and p=4 is the number of rows of the local weak observability matrix, which is the product between the number of states, including the extended states, and the number of measured outputs.

Applying Equation (26) to the system described in Equation (19), considering just the measured output voltage $u_{1}\left(t\right)$, in Equation (29), the number of columns equals the number of rows, p=n=4, which implies that just one determinant must be checked. The unique determinant is not equal to zero.

## 4. Experiment Setup

**Figure 3:**
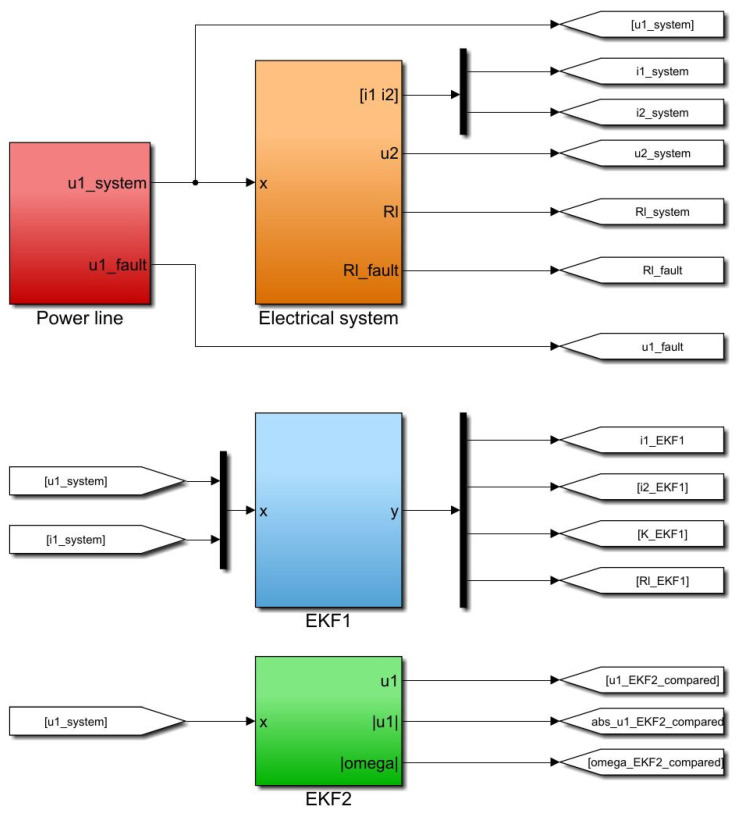
Overview of the simulation setup with the state observer EKF1 (light blue), state observer EKF2 (green), power line with implemented fault (red), and the electrical system (orange).

For the implementation and simulation of the developed state observers and the electrical system, MATLAB^®^/Simulink R2021a^®^ was used with a sampling time $T_{s}$ = 1 × 10${}^{-5}$ s. Figure 3 shows the experiment setup of the electrical system (orange) including the load resistance $R_{L}\left(t\right)$, the state observer EKF1 (light blue) for the mutual inductor, the harmonic state observer EKF2 (green), and the power line (red) with Gaussian distributed (variance = 300) output voltage $u_{1}\left(t\right)$.

A universal control transformer with $i_{1,\max}=1.45$ A and $i_{2,\max}$= 2.78 A is utilized for the experiment setup, and the implemented values for the primary and secondary inductances are $L_{1}$ = 2246 ± 1.30 mH and $L_{2}$ = 723.858 ± 0.774 mH, with the mutual inductance *M* = 1.122. The electrical serial resistances follow $R_{1}$ = 156.474 ± 0.768 Ω and $R_{2}$ = 67.079 ± 0.379 Ω. The electrical load resistance is determined to be $R_{L}$ = 181.36 Ω.

The simulation studies are based on variations of the initial resistance values through the assessment criteria defined by:

(30) $$\begin{array}{cc} J= & [\int_{t_{\mathrm{stop}}}^{t_{\mathrm{start}}}{\left(i_{1}\left(t\right)-{\hat{i}}_{1}\left(t\right)\right)}^{2}dt,\;\;\;\int_{t_{\mathrm{stop}}}^{t_{\mathrm{start}}}{\left(i_{2}\left(t\right)-{\hat{i}}_{2}\left(t\right)\right)}^{2}dt,\;\;\;\int_{t_{\mathrm{stop}}}^{t_{\mathrm{start}}}{\left(K\left(t\right)-\hat{K}\left(t\right)\right)}^{2}dt,\\ \; & \int_{t_{\mathrm{stop}}}^{t_{\mathrm{start}}}{\left(R_{l}\left(t\right)-\hat{R_{l}}\left(t\right)\right)}^{2}dt{]}^{T}\;=\;\;\;{\left[\;J_{1},\;\;\;J_{2},\;\;\;J_{3},\;\;\;J_{4}\;\right]}^{T^{{}^{{}^{{}^{}}}}}. \end{array}$$

## 5. Results and Discussion

This section shows representative results that demonstrate the proposed theory in the previous sections, with a focus on electrical features of the considered transformer. The EKF1 and EKF2 model uncertainty matrices are defined as follows:

$$\underset{Q_{\mathrm{EKF}}1}{\underset{︸}{Q_{1}}}\;=\;\left[\begin{array}{cccc} 10^{2} & 0 & 0 & 0\\ 0 & 10^{3} & 0 & 0\\ 0 & 0 & 10^{3} & 0\\ 0 & 0 & 0 & 10^{8} \end{array}\right],\;r_{1}\;=\;10^{5};\;\;\;\underset{Q_{\mathrm{EKF}}2}{\underset{︸}{Q_{2}}}\;=\;\left[\begin{array}{ccc} 0 & 0 & 0\\ 0 & 5\times 10^{-6} & 0\\ 0 & 0 & 10^{-21} \end{array}\right],\;r_{2}\;=\;10^{2},$$

the with covariance matrix for EKF1 $P_{0}^{+}=\mathrm{diag}\left[\;0\;\;0\;0\;\;0\;\right]$ and for EKF2 $P_{0}^{+}=\mathrm{diag}\left[\;0\;\;1\;\;1\;\right]$.

Process noise matrix $Q_{1}$ is set manually using a trial-and-error method. Larger values of the variance are reserved for unknown dynamics with “less measured variables”. In particular, this is the case of Gaussian mixture dynamics as K(t) and $R_{L}\left(t\right)$ dynamics. It is possible to notice that the dynamics of current $i_{1}\left(t\right)$ are the dynamics with more information. In fact, the deterministic model states a clear dynamic relation; see Equation (4). Moreover, the voltage $u_{1}\left(t\right)$ and the current $i_{1}\left(t\right)$ are directly measured and, in this sense, smaller uncertainties can be considered in the corresponding process noise. In fact, through the measurements, we can see that the uncertainties related to the current $i_{2}\left(t\right)$ and the coupling factor K(t), together with the electrical load resistance $R_{L}\left(t\right)$, weigh less than in the other dynamics. The dynamics of current $i_{2}\left(t\right)$, as in Equation (5), present a larger process noise variance because here the measured variable is just voltage $u_{1}\left(t\right)$ and, in this sense, the information on the dynamics is more affected by uncertainty due the presence of the stochastic variables K(t) and $R_{L}\left(t\right)$.

The process noise related to matrix $Q_{2}$ can be interpreted in a similar way. In fact, the first equation can be considered an equation that represents the model in an almost exact way, except for the discretization error, which, considering the adopted sampling time, is relatively small. The third equation, even though it involves Gaussian mixture dynamics, is basically the output measured variable and, thus, the uncertainty associated with this dynamic can be considered to be very small. The largest variance is related to the second dynamic, because this dynamic involves Gaussian mixture dynamics that are not measured. We can associate the largest process noise variance to these dynamics.

**Figure 4:**
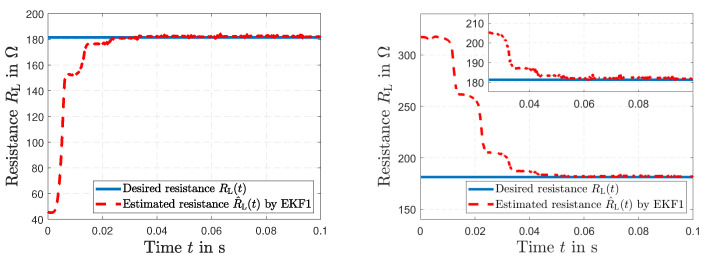
Simulated and estimated load resistance $R_{L}$(t) and ${\hat{R}}_{L}$(*t*) by EKF1 with initial condition error of −75% in $R_{L}$(t) (**left**). The same simulation under an initial condition error of +75% (**right**).

The resulting errors based on the assessment criteria in Equation (30) under different simulation conditions of the estimation states of EKF1 are presented in Table 1. Figure 4 shows how the observer can correct the initial error condition in $R_{L}$(t) of ±75% in a relatively short time.

**Figure 5:**
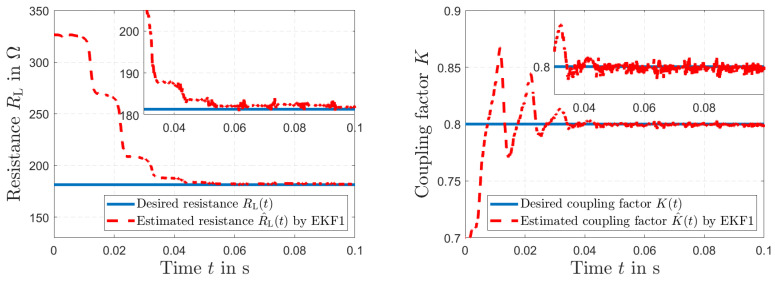
Simulated load resistance $R_{L}$(t) and estimated load resistance ${\hat{R}}_{L}$(*t*) by EKF1 (**right**); simulated coupling *K*(*t*) and estimated coupling $\hat{K}\left(t\right)$ by EKF1 (**left**).

**Figure 6:**
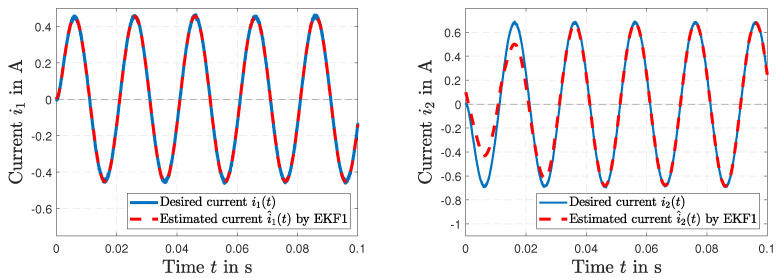
Simulated current $i_{1}$(*t*) and estimated current ${\hat{i}}_{1}\left(t\right)$ by EKF1 (**left**); simulated current $i_{2}$(*t*) and estimated current ${\hat{i}}_{2}\left(t\right)$ by EKF1 (**right**).

The simulated load resistance $R_{L}$(*t*) of the secondary side and the estimated resistance ${\hat{R}}_{L}$(*t*) by EKF1 are depicted in Figure 5 (left). We can see that the EKF1 can correct the initial error condition in $R_{L}$(*t*) of +80% in a relatively short time. Figure 5 (right) presents the simulated inductive coupling coefficient *K*(*t*) and the estimated coupling $\hat{K}$(*t*) by EKF1. Figure 6 (left) shows the desired current $i_{1}$(*t*) together with the estimated current ${\hat{i}}_{1}$(*t*) of the primary transformer winding by EKF1. An accurate tracking of the current signal can be seen. The desired current $i_{2}$(*t*) of the secondary transformer winding and the estimated current ${\hat{i}}_{2}$(*t*) by EKF1 are depicted in Figure 6 (right). After Δt=0.04 s, accurate tracking is visible.

**Figure 7:**
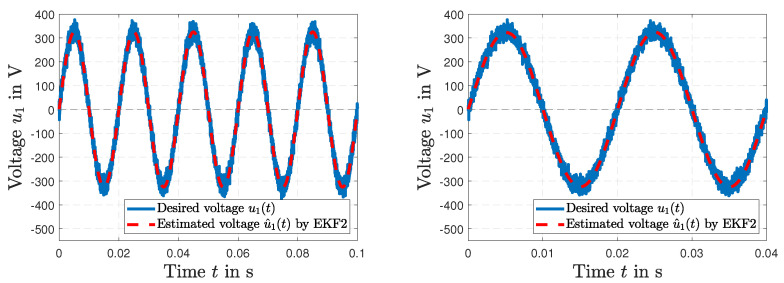
Simulated power line voltage $u_{1}$(*t*) and estimated voltage ${\hat{u}}_{1}$(*t*) by EKF2 (**left**); results of the voltage estimates by EKF2 in detail (**right**).

**Figure 8:**
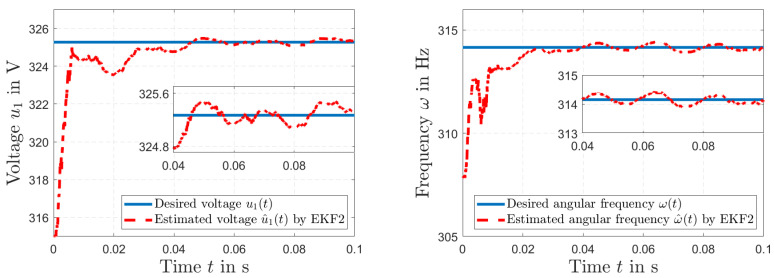
Simulated voltage amplitude of $u_{1}$(*t*) and estimated amplitude of ${\hat{u}}_{1}$(*t*) by EKF2 (**left**); simulated power line frequency ω(*t*) and estimated frequency $\hat{\omega }$(*t*) by EKF2 (**right**).

Figure 7 (left) shows the desired power line distribution across the primary winding $u_{1}$(*t*) and the simulated estimation of voltage ${\hat{u}}_{1}$(*t*) by the harmonic EKF2. Details of the results are presented in Figure 7 (right). The simulated amplitude of voltage $u_{1}$(*t*) and the estimated voltage ${\hat{u}}_{1}$(*t*) by EKF2 are depicted in Figure 8 (left), and the frequency ω(*t*) and the estimated frequency $\hat{\omega }$(*t*) by EKF2 are shown in Figure 8 (right).

**Figure 9:**
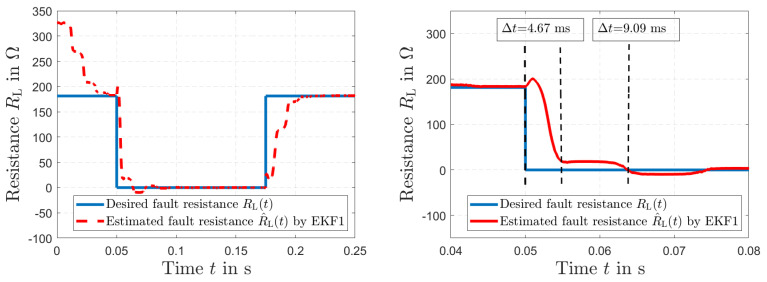
Time histories of the desired resistance $R_{L}$(*t*) and the fault tracking of the estimated resistance ${\hat{R}}_{L}$(*t*) by EKF1 (**left**), and in detail (**right**).

**Figure 10:**
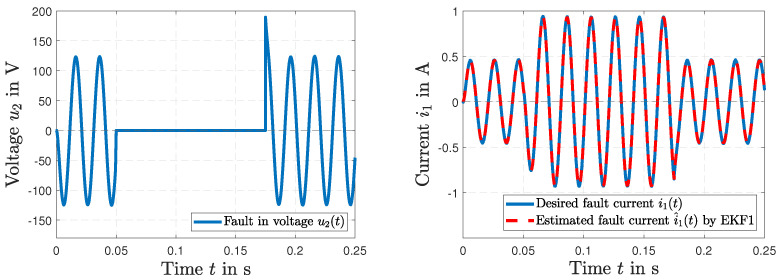
Simulated short circuit $R_{L}$(t) = 0 Ω at t=0.05 s with the fault in the voltage $u_{2}$(*t*) (**left**); the desired current $i_{1}$(*t*) with the fault tracking of the current ${\hat{i}}_{1}$(*t*) by EKF1 (**right**).

**Figure 11:**
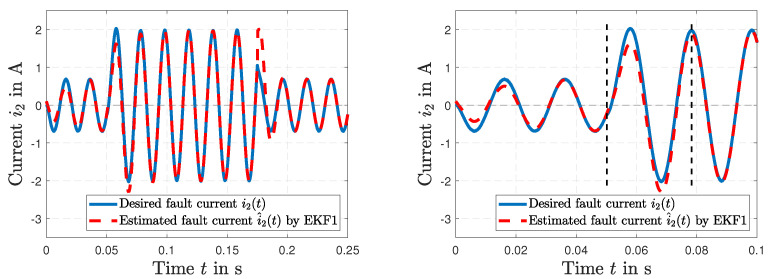
Time histories of the desired current $i_{2}$(*t*) and fault tracking of the estimated current ${\hat{i}}_{2}$(*t*) by EKF1 (**left**), and in detail (**right**).

The results of the short circuit simulation are presented in Figure 9, Figure 10 and Figure 11, where a fault occurs in the load resistance $R_{L}$(*t*) = 0 Ω at t=0.05 s. Figure 9 represents the time history of the desired resistance $R_{L}$(*t*) and the fault tracking of the estimated load resistance ${\hat{R}}_{L}$(*t*) by EKF1. After the fault occurs, a tracking in Δt=4.67 ms is visible. The resulting voltage $u_{2}$(*t*) is shown in Figure 10 (left), and the resulting current $i_{1}$(*t*) along with the current ${\hat{i}}_{1}$(*t*) estimated by EKF1 are depicted in Figure 10 (right). Two time histories of the current $i_{1}$(*t*) of the primary winding and the resulting fault tracking of the estimated current ${\hat{i}}_{1}$(*t*) by EKF1 are presented in Figure 11.

**Figure 12:**
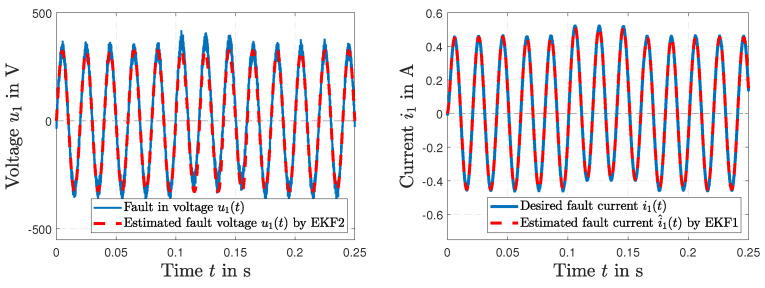
Time histories of fault tracking of the voltage $u_{1}$(*t*) of the primary winding by EKF2 (**left**); the fault tracking of the current ${\hat{i}}_{1}$(*t*) by EKF2 and the desired current $i_{1}$(*t*) (**right**).

Figure 12 presents a fault in the power line voltage $u_{1}$(*t*) with a bias of $30\sqrt{2}$ volts at t=0.1 s. The time histories of fault estimation by EKF2 are shown in Figure 12 for the voltage $u_{1}$(*t*) (left) and the estimated current ${\hat{i}}_{1}$(*t*) (right).

## 6. Conclusions and Future Work

In this paper, the connection of two EKFs for sensor fusion and EKF adaptation for fault-condition estimation were proposed. EKFs provided estimation of electrical quantities and detection of possible faults. The first EKF provided the state estimation of the galvanic decoupled secondary transformer side and the electrical load resistance. The second EKF was used for harmonic detection and estimated the amplitude and frequency of the primary winding voltage. The proposed theoretical procedures were mathematically proven and demonstrated in figures that document their efficiency. Future work will especially focus on the issues of implementing the proposed algorithms as a specific embedded system for hardware-in-the-loop prototyping, in order to increase the application possibilities for photovoltaic systems that are both a part of smart grids and separated island operations.   

## Figures and Tables

**Table 1:** Values of the criteria $J_{i}$, Equation (30), under different conditions of the load resistance $R_{L}$ by EKF1.

$R_{L}$	−75%	−50%	−25%	−5%	−1%	1%	5%	25%	50%	75%
${\hat{i}}_{1}$(*t*), $\;J_{1}$:	$1.60\times 10^{-6}$	$1.25\times 10^{-6}$	$1.08\times 10^{-6}$	$1.02\times 10^{-6}$	1.01$\times 10^{-6}$	$1.01\times 10^{-6}$	$1.01\times 10^{-6}$	$1.00\times 10^{-6}$	$1.04\times 10^{-6}$	$1.11\times 10^{-6}$
${\hat{i}}_{2}$(*t*), $\;J_{2}$:	$7.92\times 10^{-5}$	$5.60\times 10^{-5}$	$3.86\times 10^{-5}$	$4.92\times 10^{-5}$	5.62$\times 10^{-5}$	$6.04\times 10^{-5}$	$7.01\times 10^{-5}$	$1.47\times 10^{-4}$	$3.11\times 10^{-4}$	$5.45\times 10^{-4}$
$\hat{K}$(*t*), $\;J_{3}$:	$1.69\times 10^{-4}$	$9.29\times 10^{-5}$	$6.01\times 10^{-5}$	$4.92\times 10^{-5}$	$4.80\times 10^{-5}$	$4.76\times 10^{-5}$	$4.67\times 10^{-5}$	$4.57\times 10^{-5}$	$4.94\times 10^{-5}$	$5.72\times 10^{-5}$
${\hat{R}}_{L}$(*t*), $\;J_{4}$:	9.09×10	4.01×10	8.96	1.56	2.53	3.37	5.79	3.50× 10	$1.22\times 10^{2}$	$2.86\times 10^{2}$

## Abbreviations and Nomenclature

The following abbreviations are used in this manuscript:	
ARX	Autoregressive model with exogenous input
DC	Direct current
DIN	Designation of standards; German Institute for Standardization
EKF	Extended Kalman filter
FDD	Fault detection and diagnosis
IEC	Designation of standards; International Electrotechnical Commission
ISO	Designation of standards; International Organization for Standardization
KF	Kalman filter
PMSG	Permanent magnet synchronous generator
UKF	Unscented Kalman filter

The following nomenclature is used in this manuscript:	
*A*	Amplitude
$A_{F}$	Matrix of the system dynamics
F	State-input feedback matrix
$f(x\left(t\right),\;u_{1}\left(t\right))$	Nonlinear state function
$u_{1}$, $u_{2}$	Electrical voltages across the primary and secondary winding, respectively
$i_{1}$, $i_{2}$	Electrical currents of the primary and secondary winding, respectively
$L_{1}$, $L_{2}$, *M*	Primary, secondary, and mutual inductances of transformer, respectively
$L_{f}^{m}$	Lie derivatives
$u_{s}$	White noise variable
$U_{d}$	Pre-compensation matrix
$h_{2}$	Scalar output measurement function
$H_{1},H_{2}$	Output Jacobian measurement matrix, respectively
*J*	Assessment criteria
$J_{k}$	Jacobian matrix
*K*	Inductive coupling coefficient
k	Kalman gain
O(x(t))	Nonlinear observability matrix
N	Natural numbers
$N_{d}$	Number of possible determinants
p,n	Numbers of columns and rows of observability matrix, respectively
$P_{k}^{+},\;P_{k}^{-}$	Posterior and a priori estimation error covariance matrix
$Q_{\mathrm{EKF}1},\;Q_{\mathrm{EKF}2}$	Covariance matrices $Q_{1},\;Q_{2}$ of process noise, respectively
$r_{1},\;r_{2}$	Variance matrices of soft sensors
$R_{1},\;R_{2},\;R_{L}$	Primary and secondary winding and electrical load resistances, respectively
$t,\;t_{\mathrm{start}},\;t_{\mathrm{stop}}$	Continuous time, start time, and stop time, respectively
$T_{s}$	Sampling period
x(t)	System state
${\hat{x}}_{k}^{+}$	A posteriori estimation of system state
${\hat{x}}_{k}^{-}$	A priori estimation of system state
ω	Angular frequency
ϕ	Angular phase
$\phi_{1},\;\phi_{m}$	Primary stray flux and core flux of transformer, respectively
$\Phi_{s}$	Phase noises

## Appendix A

**Listing A1.** Observability test for EKF1.

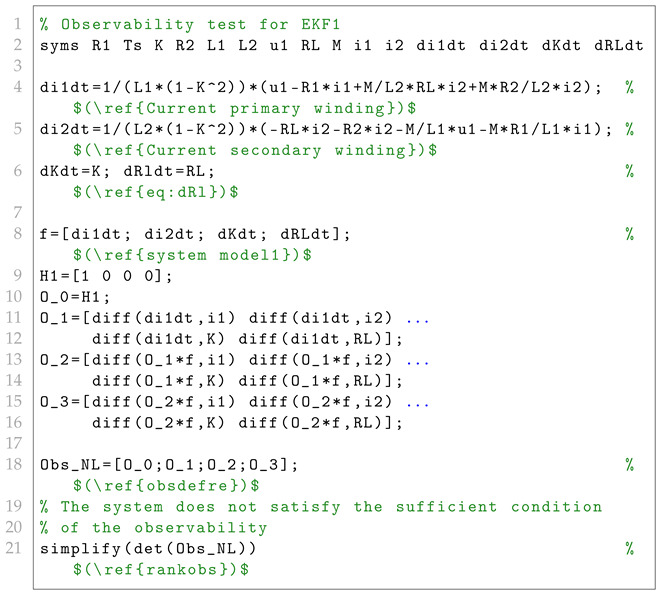

**Listing A2.** Observability test for EKF2.

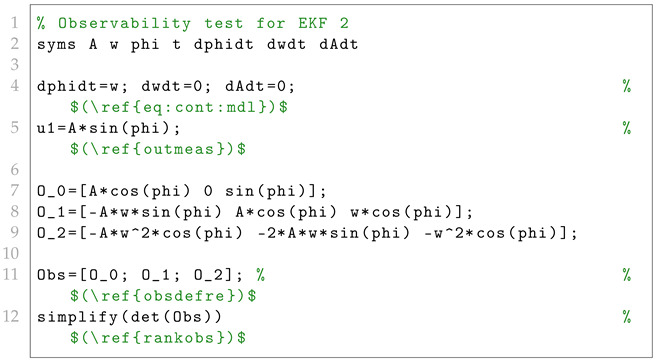

**Remark** **A1.**
*The following condition:*

(A1)
$$\det\left(\mathrm{Obs}\right)=-A{\left(t\right)}^{2}\omega {\left(t\right)}^{2}\left(\cos{\left(\phi \left(t\right)\right)}^{3}-3\sin\left(\phi \left(t\right)\right)+\sin{\left(\phi \left(t\right)\right)}^{3}\right)=0$$

*admits A(t)=0 and ω(t)=0 as solutions. Concerning the values of ϕ(t), which solve Equation (A1) using the Symbolic Math Toolbox™ by MATLAB^®^ R2021a, it follows that:*

(A2)
$$\begin{array}{c} \mathrm{solve}(\left(\cos{\left(\mathrm{phi}\right)}^{3}-3*\sin\left(\mathrm{phi}\right)+\sin{\left(\mathrm{phi}\right)}^{3}\right)=0,\mathrm{phi})\\ \;\;\;\;\;\;\;\;\;\;\;\;\;\;\;\;\;\;\;\;\;\;\;\;\;\;\;\;\;\;\;\;\;\;\;\;\;\;\;\;\;\;\;\;\;=-\log(\mathrm{root}\left(z^{6}+z^{4}*\left(6+3i\right)-z^{2}*\left(3+6i\right)-1i,z,1\right))*1i. \end{array}$$

*Two isolated real values of ϕ(t) are obtained as solutions using the following command:*

(A3)
$$\mathrm{vpa}(\log\left(\mathrm{root}\left(z^{6}+z^{4}*\left(6+3i\right)-z^{2}*\left(3+6i\right)-1i,z,3\right)\right)*1i)=2.83\;\mathrm{rad}$$

(A4)
$$\mathrm{vpa}(\log\left(\mathrm{root}\left(z^{6}+z^{4}*\left(6+3i\right)-z^{2}*\left(3+6i\right)-1i,z,4\right)\right)*1i)=-0.303\;\mathrm{rad}.$$

*Conditions A(t)=0 and ω(t)=0, together with the two isolated points ϕ(t)=2.83rad and ϕ(t)=−0.303rad, in which the sufficient observability condition is not satisfied, do not represent dynamic relevant conditions.*
